# Convergent adaptation of cellular machineries in the evolution of large body masses and long life spans

**DOI:** 10.1007/s10522-017-9713-9

**Published:** 2017-06-01

**Authors:** Eleonora Croco, Silvia Marchionni, Gianluca Storci, Massimiliano Bonafè, Claudio Franceschi, Thomas D. Stamato, Christian Sell, Antonello Lorenzini

**Affiliations:** 1 Department of Biomedical and Neuromotor Sciences, University of Bologna, Bologna, Italy; 2 Department of Specialised Experimental, and Diagnostic Medicine, University of Bologna, Bologna, Italy; 3 IRCCS Institute of Neurological Sciences, Bologna, Italy; 4 Interdepartmental Center “L. Galvani”, University of Bologna, Bologna, Italy; 5 Lankenau Institute for Medical Research, Wynnewood, PA USA; 6 Department of Pathology, Drexel University College of Medicine, Philadelphia, PA USA

**Keywords:** Body size, Aging, Longevity, Convergent evolution, Genomic stability, DNA damage, Replicative senescence

## Abstract

In evolutionary terms, life on the planet has taken the form of independently living cells for the majority of time. In comparison, the mammalian radiation is a relatively recent event. The common mammalian ancestor was probably small and short-lived. The “recent” acquisition of an extended longevity and large body mass of some species of mammals present on the earth today suggests the possibility that similar cellular mechanisms have been influenced by the forces of natural selection to create a convergent evolution of longevity. Many cellular mechanisms are potentially relevant for extending longevity; in this assay, we review the literature focusing primarily on two cellular features: (1) the capacity for extensive cellular proliferation of differentiated cells, while maintaining genome stability; and (2) the capacity to detect DNA damage. We have observed that longevity and body mass are both positively linked to these cellular mechanisms and then used statistical tools to evaluate their relative importance. Our analysis suggest that the capacity for extensive cellular proliferation while maintaining sufficient genome stability, correlates to species body mass while the capacity to correctly identify the presence of DNA damage seems more an attribute of long-lived species. Finally, our data are in support of the idea that a slower development, allowing for better DNA damage detection and handling, should associate with longer life span.

## Introduction

Multiple theories have been proposed to explain the aging process and several of them have collected, with time, significant experimental supports (López-Otín et al. 2013). In Biogerontology there is a growing appreciation that the process of aging is a multifactorial process and its impact may vary greatly among species (Gladyshev 2013). This may be because evolutionary processes have led to multiple strategies to deal with spontaneous molecular damage, both extrinsic and intrinsic. Extrinsic damage may be due to UV radiation, for example, while intrinsic damage may be due to DNA fork collapse or other molecular malfunctioning of the basic biological machinery within the cell. The different efficiencies of these strategies could explain the different rate of aging observed across species (Jones et al. 2014) and why aging is not a universal process (Bilinski et al. 2016).

## Convergent evolution in the evolution of longevity and body mass

**Fig. 1:**
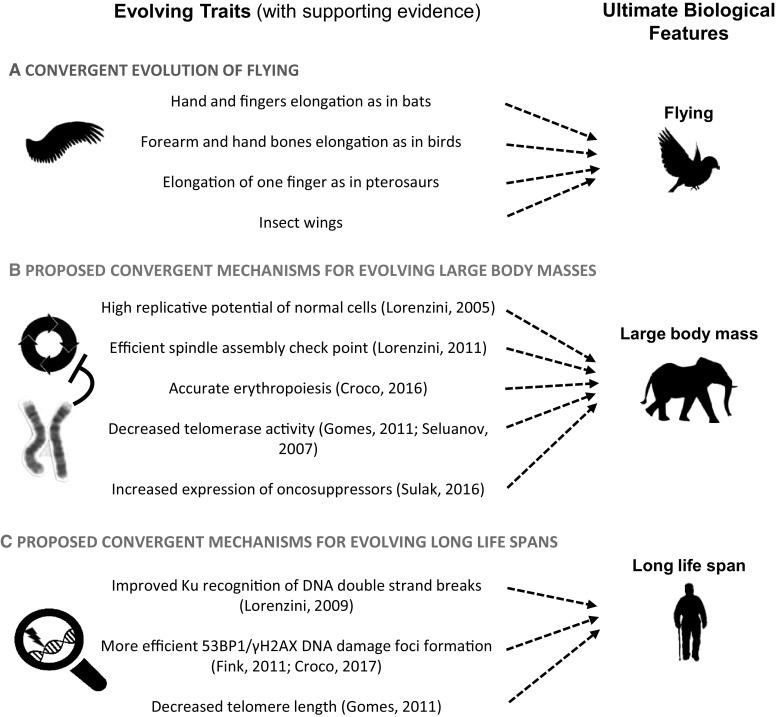
Convergent evolution. **a** Convergent evolution of flying: bird, bats, pterosaurs and insects have clear anatomical differences in their wing structure but they all evolved flying. **b** Different cellular machineries may have been optimized during the independent evolution of large body masses. **c** Different cellular machineries may have been optimized during the independent evolution of long life spans. In parenthesis are reported some key supporting references. See text for in depth description

**Fig. 2:**
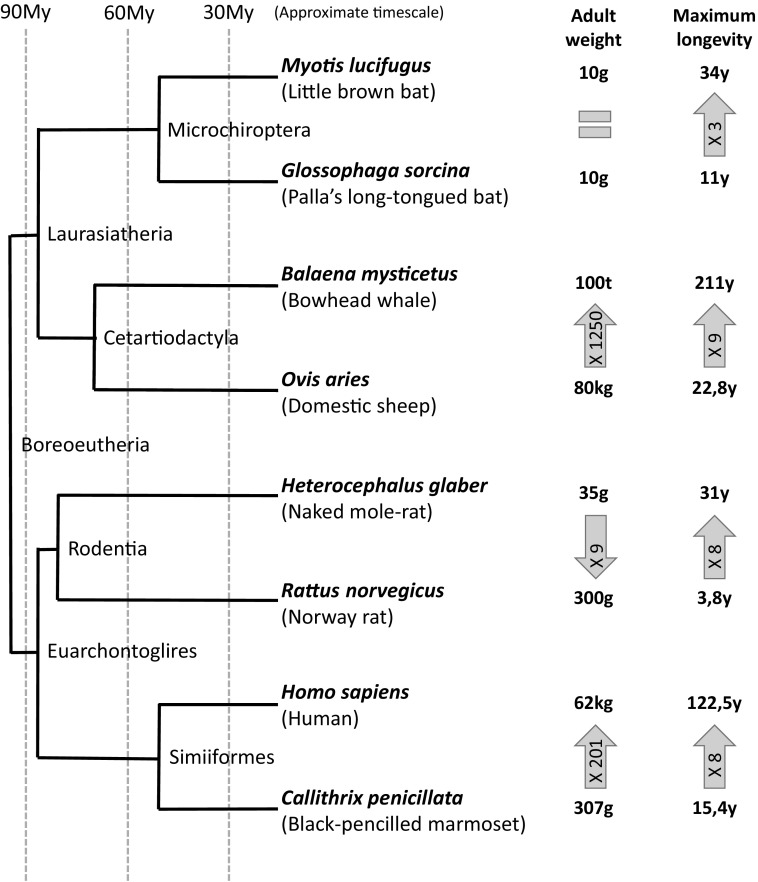
Large body sizes and significant longevities evolved multiple times. The numbers inside the *arrows* indicate the fold difference in maximum longevity and adult weight (from the smaller to the higher value). The Phylogenetic tree was built following phylogenetic information in Gomes et al. 2011; Richards et al. 2010; Jameson Kiesling et al. 2015. Maximum longevity and adult weight were taken from the AnAge database (Tacutu et al. 2013). Time scale is given only as a reference and it is not meant to be accurate (*My, * millions of years ago)

The theory of convergent evolution suggests that analogous structures, that have similar form or function but were not present in the last common ancestor, evolve independently in two or more species. Birds and bats, for example, which do not have a common winged ancestor, have both evolved wings although with very different anatomical adaptation of the upper limb (see Fig. 1, section A). Many basic cellular mechanisms are highly conserved due to the fact that life on the planet has had the form of individual cells for the majority of evolutionary time (Cooper 2000). In evolutionary terms, the mammalian radiation is a relatively recent event. If we consider longevity has evolved multiple times (see Fig. 2), one may posit that longevity of phylogenetically distant species represents convergent evolution. It seems reasonable then to look at the mammalian evolutionary tree searching if conserved cellular properties show some convergent adaptation to long life. Here we will analyze those listed in Fig. 1, sections B and C.

The DNA damage accumulation theory of aging suggests that “hits” to the genome may be responsible of the aging process (Szilard 1959). Support of this theory comes in part from the fact that several human premature aging syndromes are characterized by defects in single genes coding either for DNA repair proteins or for lamin A, a protein primarily involved in nuclear architecture with the capacity to influence genomic stability. These syndromes dramatically shorten longevity in affected patients (reviewed in Freitas and de Magalhães 2011). Based on the DNA damage accumulation theory, we might suggest that convergent evolution could be involved in increasing genomic stability in long-lived species.

Large body size and long life span have evolved multiple times in independent branches of the phylogenetic tree of life. When considering mammals, for example, starting from a common squirrel sized ancestor (O’Leary et al. 2013) which was probably short lived, there are currently species that range in body mass from few grams to more than hundred tons and range in lifespan from a few years to hundreds of years. Additionally, although these two features are positively associated (De Magalhães et al. 2007) they are not strictly linked: see few clear examples in Fig. 2. Omitting for a moment the DNA damage theory of aging, we can hypothesize that convergent evolution in cellular machineries could also play a role in the evolution of large body masses. It is reasonable to expect, in fact, that large species, having more cells, would be at higher risk of cancer if, with the increase in size, genomic stability mechanisms are not concurrently improved (Peto 2015).

With these considerations in mind, we have searched for cellular characteristics that have the potential to provide longevity assurance across mammalian species that vary in adult size and in longevity. To date, our laboratories, have analyzed: (1) the capacity of somatic tissue for extensive proliferation while maintaining a normal diploid chromosome content, (2) telomere length and (3) the capacity to detect DNA damage at the molecular level. In this review, the focus is this body of work and work strictly related to these subjects from other authors. For this reason, we ask forgiveness if this review is not comprehensive of all relevant literature.

## Cellular characteristics possibly related to the evolution of large body masses

**Fig. 3:**
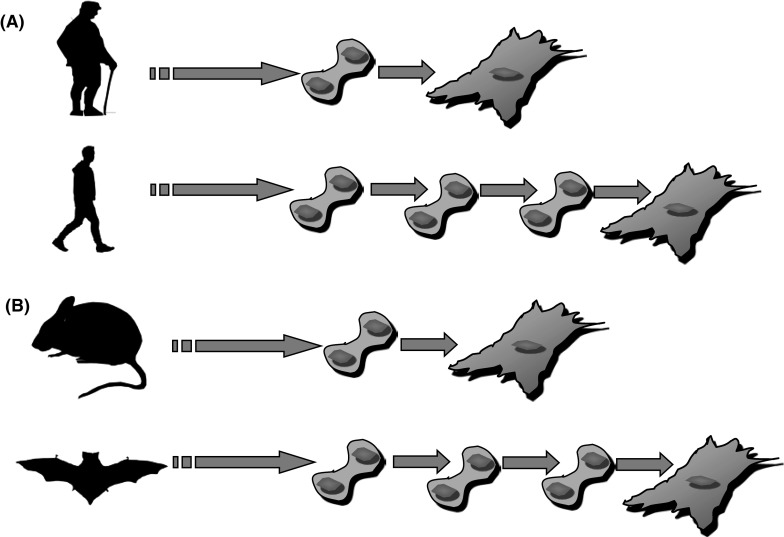
Two expectations from the hypothesis that the maximum number of cellular divisions in vitro relates to species longevity. **a** Proliferation competent cells from old donors are expected to accomplish less cell division than cells from young donor. **b** Proliferation competent cells from short-lived species are expected to accomplish less cell divisions than cells from long-lived species (e.g., house mouse max. longevity is 4 years; little brown bat max. longevity is 34 years; max longevity from the AnAge database Tacutu et al. 2013)

 Hayflick and Moorhead demonstrated that somatic cells have a limit in the number of cell divisions they can achieve (Hayflick and Moorhead 1961), contradicting the preexisting paradigm according to which cells isolated from the body have an unlimited replicative capacity (Carrel 1912). This proliferative limit will be later referred to as the “Hayflick limit” (for a recent literature revision of this model system see a book edited by Rattan and Hayflick 2016). Hayflick also suggested that this limit is a general characteristic of all normal diploid cells and that unlimited replicative capacity is a characteristic of cancer cells (abnormal cells). Another suggestion was that this limited replicative capacity represents aging at the cellular level. The idea that limited replicative capacity is “pro-aging” fits very well with the modern understanding of the biology of non-aging animals. Unlimited proliferative capacity of totipotent/or pluripotent stem cells, in fact, seems to be the key to immortality of some Porifera and Cnidarians (Petralia et al. 2014). Additionally this idea has two obvious and testable implications: the first is that cells from younger individual are expected to be able to accomplish more cell divisions than cells from old individuals; the second is that cells from long-lived species are expected to be able to accomplish more cell divisions than cells from short-lived species (Fig. 3).

Regarding the first implication, much research has been performed, almost exclusively, evaluating replicative capacity in culture because of the scarcity of reliable techniques that will allow tracking replicative capacity of cells in vivo. This literature has been revised in one chapter of the above mentioned book (Lorenzini and Maier 2016). In this assay, the authors concluded that the first implication is not supported, at least for skin fibroblasts that are the cell type mainly used for this kind of studies. For the sake of completeness, we should mention that in vitro colony forming ability could be related somehow to donor age (also reviewed in Lorenzini and Maier 2016). A relationship that could depend from a decrease in the speed of doubling with increasing donor age. Decrease that has been observed in human skin fibroblasts (Kalfalah et al. 2015) and human retinal pigment epithelium cells (Flood et al. 1980).

The second implication is discussed in details in the following paragraph.

### Replicative capacity

Regarding the relationship between replicative capacity (measured as number of cell doublings) and species longevity less research is available. Stanley and coworkers, measuring replicative capacity of ten cell strains each from a different mammalian species, concluded that there is not relationship between replicative capacity and longevity (Stanley et al. 1975). Röhme, on the contrary, measuring 36 cell strains from eight mammalian species reports a significant correlation (Röhme 1981). A larger study by Lorenzini et al. on 59 cell strains from 11 mammalian species that used only adult derived skin fibroblasts (while previous studies mixed strains from different developmental stages and tissues), concluded that although replicative capacity and longevity are positively related, the major determinant of replicative capacity is species adult body mass, not longevity (Lorenzini et al. 2005). In other words, the cells from large species are the ones capable of sustaining multiple rounds of division while preserving a normal karyotype. Moreover, they are able to sustain proliferation to a greater extent than cells from long-lived but small sized species.

It is known that oxygen negatively affect in vitro replicative life span (Balin et al. 2002) and one criticism, that relates to all of the data discussed above, is that cells from some species (e.g. mouse) appears not to display replicative senescence when cultured under low oxygen (Parrinello et al. 2003; Gomes et al. 2011). Accepting this criticism, we may reformulate the above finding stating that replicative capacity under stressful ambient oxygen conditions correlate primarily with species adult body size than to longevity.

Gillooly et al. report data suggesting that the positive relationship between replicative capacity and body mass may be valid also for birds, reptiles, amphibians and fishes (Gillooly et al. 2012). The observations that telomere loss is minimal in skin of old donors [(Krunic et al. 2009) and references therein] and their fibroblasts retain a high in vitro replicative capacity (Maier et al. 2007) may be considered in support of this view. Conversely, the cells derived from the biopsies of taller nonagenarians had less residual replicative capacity compared with the cells derived from the shorter ones (Maier et al. 2008). In other words, the requirement to cover the larger surface of a taller body during development leave the cells in the adult with less residual proliferative capacity. If the number of potential cell divisions was related to longevity, one would predict that taller individuals (the one with less residual replicative capacity) should be the short-lived ones. This appears not to be the case; see for example, a recent large meta-analysis showing a positive association between stature and longevity (NCD Risk Factor Collaboration 2016).

As already mentioned, body mass and longevity are loosely and positively linked among species. The simplest explanation for this relationship is that even with very fast proliferation rates, very large bodies will require longer developmental time than small bodies since every organism starts from a single cell. The complex issue regarding the intraspecies relationship between body size and longevity, marginally addressed above, is not the focus of this review and is discussed in more depth elsewhere (Bartke 2012; Lorenzini 2014; Samaras 2012).

### Spindle assembly checkpoint fidelity

The spindle assembly checkpoint blocks progression into telophase if the chromosomes are not correctly aligned at the mitotic spindle equator (Musacchio and Salmon 2007). We have performed a small investigation on the efficiency of this checkpoint on six mammals using cultured fibroblasts and the results support our above stated view (Lorenzini et al. 2011a). We have observed that the efficiency of this checkpoint is higher in large species, while species longevity does not appears as a key factor. Although this analysis suggests that an increased efficiency of the spindle assembly check point could be more important for evolving large body size than longevity, this does not exclude the possibility that aneuploidy may negatively affect life span as well. Baker et al. in fact, report that mice with low levels of the spindle assembly checkpoint protein BubR1, display progeroid features: dwarfism, lordokyphosis, and short lifespan (Baker et al. 2004).

### Spontaneous in vivo frequencies of micronucleated erythrocytes

Additional support of our hypothesis comes from a meta-analysis we have performed on the spontaneous in vivo frequencies of micronucleated erythrocytes. The spontaneous appearance of micronuclei in circulating erythrocytes is a sign of poor mitotic efficiency during erythropoiesis. Collecting data from eight pre-existing studies we have found that the frequency of micronucleated erythrocytes correlates primarily with body mass rather than longevity (Croco et al. 2016). We should note that this is a negative correlation. These data suggest that, although both large bodies and long-lived bodies require the production of a large number of erythrocytes by the bone marrow, size is the most important constraining factor requiring a more efficient cellular machinery for replicating the genome.

## Cellular characteristics possibly related to the evolution of long life spans

The capacity to detect molecular damage is a potential cellular determinant of longevity. Detecting the presence of damage is a necessary step in order to proceed with repair or, if the damage is irreparable, with other means of damage control, as the induction of cellular senescence or apoptosis. Because of its hierarchical role, the most serious molecular damage is DNA damage. Among the different types of DNA damage, the most harmful lesions are double-strand breaks (DSBs). Two major repair pathways have evolved to repair DSBs: non-homologous end-joining (NHEJ) and homologous recombination (HR). NHEJ is the repair mechanism responsible for repairing the majority of DSBs in mammals (Kakarougkas and Jeggo 2014). This is because NHEJ is active throughout the cell cycle, whereas HR is typically active only during the S and G2 phases, and the majority of cells in the adult body are in G0 (Rothkamm et al. 2003). Additionally, NHEJ is significantly faster than HR (Mao et al. 2008). Intraspecies observations support a role of NHEJ in cancer and aging. In humans, a decrease in DNA end-joining capacity is associated with an increase in breast cancer risk (Bau et al. 2007). In mice, double mutants, as well as single mutants of either component of the Ku70/Ku80 protein heterodimer (a key component of NHEJ), display an approximate 66% reduction in life span, with signs of early aging (Li et al. 2007).

### DNA-end binding

**Fig. 4:**
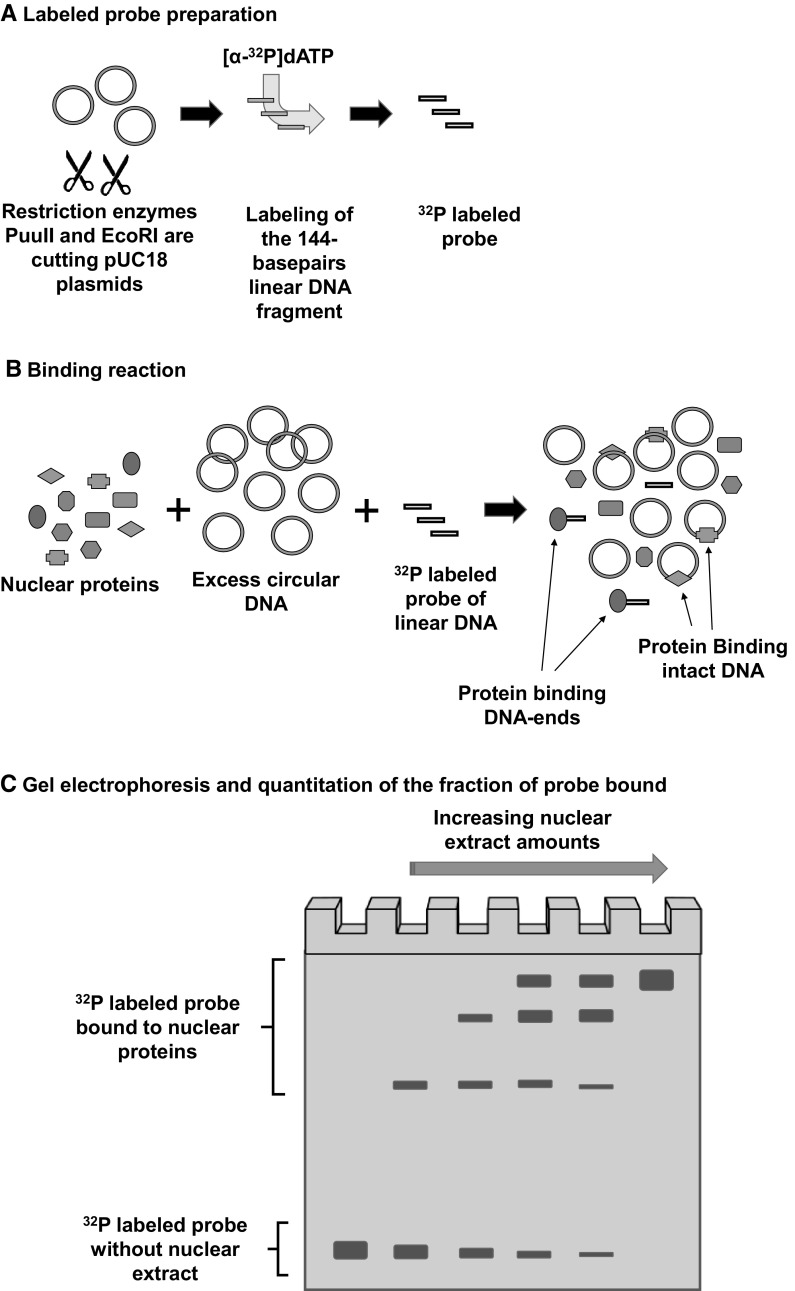
DNA-end biding assay. **a** A pUC18 plasmid is digested with both PuuII and EcoRI restriction endonuclease enzymes to generate a 144-base pair probe, then the linear probe is labeled with ^32^P. **b** Competitive binding between protein nuclear extract, excess unlabeled circular plasmids and labeled linear probes. **c** Different mixtures, containing constant amounts of unlabeled plasmid and labeled probe, but increasing amounts of nuclear extracts, are separated on a 5% polyacrylamide gel at 20–25 mA. The slowing down in the probe migration (shift) is evidence of the formation of complexes between DNA-ends and proteins. DNA-end binding activity is the amount of protein required to bind 50% of the probe. This amount is obtained by interpolation in a plot displaying “fraction of probe bound” versus “protein amounts” (Getts and Stamato 1994)

Stamato and colleagues have developed a method to study the initial step of NHEJ: the recognition of double stranded DNA breaks (or DNA ends) by nuclear proteins, a property that is strictly ascribable to the Ku heterodimer (Getts and Stamato 1994). This method consists of two steps. The first step is based on a competitive binding between a nuclear proteins extract, a ^32^P labeled linear DNA probe and an excess of unlabeled circular plasmid DNA containing the same sequence of the linear DNA probe as competitor. Since the circular plasmid is in large excess (1000-fold ratio competitor to probe), nuclear proteins with affinity for particular sequences, present in the probe, will bind the excess circular plasmid and only the protein with affinity for DNA-end will bind the labeled probe. The second step consist of an estimation of the amount of binding complexes by measuring the proportion of shifted probe on a southern blot (Fig. 4).

Using this assay, Lorenzini et al. have measured the capacity to bind DNA-end in cultured fibroblasts from 12 different mammalian species. The results show a striking, positive relationship between DNA-end binding and species longevity (Lorenzini et al. 2009). What makes this relationship interesting is the lack of a correlation between DNA-end binding activity and species adult body mass. In fact, other mechanisms of genomic surveillance such as poly(ADP-ribose) polymerase (PARP) activity (Grube and Bürkle 1992) and the repair of UV induced DNA lesions (Hart and Setlow 1974; Cortopassi and Wang 1996), positively correlates both with longevity and body mass (see dedicated reanalysis in Promislow 1994 and Stuart et al. 2013). Thus, the end-binding activity appears to be more closely related to longevity than other mechanisms of genomic surveillance.

We proposed that the different abundance of the first proteins involved in NHEJ (the Ku heterodimer and DNA-PKcs) might likely explain the different binding activities (Lorenzini et al. 2009). In support of this hypothesis, the genes XRCC6 that codifies for Ku70, together with the gene NHEJ1 that codifies for XLF (another protein involved in NHEJ), have been found more expressed in human and in the naked mole rat (a very long-lived rodent) in comparison with mice (White and Vijg 2016).

### DNA damage nuclear foci

Convenient markers for the presence of DSB are the phosphorylation of the histone variant H2AX (γ-H2AX) and the accumulation of the 53BP1 protein in distinct nuclear foci (Nikolova et al. 2014). Both of these events can be assayed by immunofluorescence. Since these markers of genomic damage can be measured with ease, they are largely used in basic science and their use could move to the clinic in the near future (Somaiah et al. 2016).

#### DNA damage foci and intraspecies longevity

Kalfalah et al. report a significant increase in γH2AX abundance in untreated skin fibroblasts from 15 woman spanning the age spectrum 20–67 years (Kalfalah et al. 2015), measurements that significantly correlated with structural chromosome aberrations. Waaijer et al. report a small but highly significant increase in 53BP1 foci abundance per nucleus in untreated skin fibroblasts derived from 100 patients spanning the age spectrum 20–90 years (Waaijer et al. 2016). These two and other analogous reports, see for example (Orta and Günebakan 2012) may be considered supporting the theory of DNA damage accumulation being causative for the aging process.

At this regard, 53BP1 protein up-regulation was observed in human fibroblasts isolated from long living subjects (centenarians) compared to young and old donors, in absence of any changes of the phosphorylated active form of 53BP1 (Lattanzi et al. 2014). Notably, in cells from centenarians exposed to oxidative stress a higher level of phosphoactive-53BP1 nuclear foci accumulation and their rapid clearance compared to younger controls was observed. The phenomenon was paralleled by a lower basal level of the DNA damage sensor γH2AX (which likely masters HR, Storci et al. in preparation) compared to fibroblasts from old subjects. The above described scenario in centenarian fibroblasts is reminiscent of literature data reporting a repression of HR repair in in vitro replicative senescence giving the way to the 53BP1 dependent mechanism (Mao et al. 2012). Regarding to this issue it is known that 53BP1 is capable to hamper HR response (Bunting et al. 2010). Notwithstanding this evidence, the up-regulation of NHEJ recombination pathway in centenarians represents a reshaping of the DNA damage response rather than a decline in the DNA repair capability (Lattanzi et al. 2014) (Storci et al. in preparation). It has been hypothesized that γH2AX mediated recruitment of 53BP1 to DNA damage nuclear foci could involve methylated histone residues (FitzGerald et al. 2009). At this regard, it is necessary to take into account that histone methylation status affects aging of adult stem cells in which DNA damage repair is a pivotal driver of tissue aging and dysfunction (Pollina and Brunet 2011). Consequently, it is necessary to deepen our understanding of the role of 53BP1 in the different modalities of NHEJ (see discussion and Ciccia and Elledge 2010) and more studies are needed to unravel the links between epigenetic regulation, genomic stability, NHEJ and human longevity.

#### DNA damage foci and interspecies longevity

We tested whether, even by this other mean to respond to genotoxic damage, long-lived species have more efficient cellular machineries. To this end, we have exposed skin fibroblasts derived from different mammals to the same concentration of two genotoxic agents (etoposide and neocarzinostatin). We found that long lived species display higher abundance of DNA damage nuclear foci (Fink et al. 2011; Croco et al. 2017). Since these markers are used to study the presence of DNA damage and a higher presence of these markers is usually interpreted as a higher presence of damage, we also evaluated DNA damage using direct assays such as the comet assay and the micronucleus assay. The first assay measures DNA fragmentation while the second measures if a whole chromosome or a fragment has been separated from the rest of the genome during mitosis. Our analysis has revealed that longer-lived species display both a lower level of DNA damage (measured by these two direct assays) relative to shorter–lived species, and a higher presence of DNA damage nuclear foci. These results suggest a lower level of damage coupled with an enhanced damage response. This is consistent with the DNA-end binding data.

### To preserve genome stability is there a need for more proteins, better proteins, or both?

The variation, we have observed, in the ability to bind DNA-ends is extremely large: 100 fold from mouse to human. In addition, the difference in abundance of the proteins directly involved in DNA-end binding, Ku80 and DNA-PKcs, is also on the order of a 100 fold difference. Thus the differential abundance could, in itself, explain the difference in DNA-end binding activity (Lorenzini et al. 2009).

The hypothesis that specific cellular functionalities, necessary to guarantee genomic stability, may be upregulated simply by increasing the abundance of key proteins it is a straightforward one. Sulak et al. for example, have shown that the Asian and African elephants (among the largest but also among the longest-lived mammals species), encodes up to 19 additionally gene copies of TP53 (Sulak et al. 2016). This ensues in an increased expression of the oncosuppressor following genotoxic stressors and a consequent increase in apoptosis in comparison to phylogenetically closely related species (hyrax, aardvark, and armadillo) that are much smaller and have only one or two copies of TP53.

We have observed that the expression of 53BP1 is higher in long lived species compared to short lived ones and that long lived species have also a higher capacity to form DNA damage nuclear foci (Fink et al. 2011; Croco et al. 2017). The magnitude of these differences, although statistically significant, is small. It is plausible, then, to hypothesize that also protein structural variation could play a role in the evolution of longevity and body mass. For example, Parp activity varies more than fivefold from short lived to long-lived mammals. Yet, Parp protein abundance across species is not related to its activity level (Grube and Bürkle 1992). In our analysis of proteins involved in NHEJ, in addition to Ku80 and DNA-PKcs we also have examined DNA ligase IV using an antibody raised against a peptide conserved between human and rodents and we found that abundance for this protein is similar among human, hamster, and mouse cells (Lorenzini et al. 2009).

When one considers these examples, it appears that evolutionary changes to improve genomic stability can do so by structural modification (which can improve protein functionality) or by the overexpression of key proteins involved in DNA repair, cell cycle control, induction of apoptosis and senescence. Although it will be difficult to disentangle the different aspects of these evolutionary processes, we believe it will be highly informative to consider the role of many molecular mechanisms rather than focusing on a single alteration as a driver of genomic stability.

## Cellular characteristics possibly related to the evolution of both large body masses and long life spans: telomere biology

Since it has become clear that, in most cases, what it is limiting replicative capacity in vitro is telomere shortening (Bodnar et al. 1998), telomere biology has been deeply investigated.

Intraspecies investigations have shown the intricacy of telomere biology: for example telomere length is uneven within individuals and tissues (Leufke et al. 2014); telomerase activity is tissue context sensitive (Krunic et al. 2009) and replication is not the only mechanism of telomere attrition (von Zglinicki 2002). Notwithstanding this complexity, comparative investigations in cell culture systems have shown fundamental differences among mammals (Seluanov et al. 2007; Lorenzini et al. 2009; Gomes et al. 2011). These works, summarized in Stuart et al. (2013), clearly show that short telomeres and low or absent telomerase activity are characteristics of long-lived and large species and the opposite is true in short-lived and small species. Since is now clearly established that telomere shortening can exert a tumor-suppressive effect (Maciejowski and de Lange 2017), one may suggest that convergent evolution has led to the development of short telomeres and absence of telomerase activity to limit tumor growth. Larger species, in fact, have more cells and thus more targets for transformation. In addition, long-lived species have more time to accumulate tumor predisposing mutations. In their analysis, Gomes et al. suggest that shutting down telomerase activity in adult tissue seems a strategy adopted for evolving large body mass, while adopting short telomeres a strategy adopted for evolving long life spans (Gomes et al. 2011). These implications of longevity and size are poorly appreciated. Peto has estimated that the risk to develop a tumor per gram of tissue is 3 trillion times lower in humans than in mice (Peto 2015).

## Conclusions

We propose that DNA damage recognition represents an evolutionarily convergent longevity assurance mechanism. DNA can be damaged in several ways (Freitas and de Magalhães 2011) and, potentially, the capacity to recognize all different types of damage may contribute to species longevity. Our data are supportive for a role of the capacity to recognize DSBs, whose accumulation could be particularly relevant in driving aging (White and Vijg 2016). Our data also point to a potential key role of NHEJ. The commonly accepted idea is that NHEJ is more error-prone compared with HR; the issue, although, is probably more complex. NHEJ includes canonical NHEJ (C-NHEJ) and alternative EJ (A-EJ), characterized by ends-resections and independent from Ku80. Additionally A-EJ could represent more processes that are actually distinct (Charbonnel et al. 2011). One possibility is that C-NHEJ is indeed not an error prone mechanism while A-EJ is (Bétermier et al. 2014). We could then speculate that evolving mechanisms, that favor C-NHEJ to the detriment of A-EJ, are a way toward gaining increased longevity. A hypothesis that will deserve further testing.

It appears that improved genomic stability has evolved in multiple phylogenetic lineages through multiple mechanisms. This view of evolutionary adaptation in the DNA repair capacity as a determinant of longevity is consistent with our previous discussions on this relationship. For example, we have previously proposed that, once damage has been recognized, cells require time to adopt the best strategy forward: repair the damage or induce senescence or apoptosis in order to select away irreparable cells. Therefore, the ability to devote time to repair may be a key aspect of long-lived species (Lorenzini et al. 2011b). If time for repair is, indeed, a key resource for the organism, one would predict that developmental rate would be related to longevity. Development is the life period when rapid cell divisions are essential and a time during which unrepaired DNA damage occurring in a single cell, can clonally expand to all its descendants, amplifying its negative burden. In support of this vision, Ridgway et al. analyzing 50 species of bivalves, report an inverse relationship between growth rate and longevity (Ridgway et al. 2011); this report was further supported by a following study which included a data set containing 297 bivalves species (Moss et al. 2016). Additionally a correlation between maximum adult life span and time to maturity (defined as gestation or incubation time plus age at sexual maturity) is reported for 606 mammals and 69 birds species (Magalhães et al. 2007). The authors, in addition, have considered the bias represented by body mass in this correlation. After the appropriate analysis, their conclusion remains that “developmental time is strongly associated with maximum adult life span”. Finally, one experimental proof is available for this growth rate—lifespan trade-off in the three-spined sticklebacks fish (Lee et al. 2013).

In conclusion, we suggest that a higher capacity to recognize DNA damage may benefit a developing organism allowing for the buildup of an adult body free of molecular damage and consequently long lasting. The role of DNA damage recognition and repair in the adult life may be similar, allowing for an efficient cellular turnover in tissues but also potentially different. Cells that over react to molecular damage, for example, could result detrimental contributing to “Inflammaging” (Franceschi et al. 2016).
